# Identification and integrated analysis of differentially expressed lncRNAs and circRNAs reveal the potential ceRNA networks during PDLSC osteogenic differentiation

**DOI:** 10.1186/s12863-017-0569-4

**Published:** 2017-12-02

**Authors:** Xiuge Gu, Mengying Li, Ye Jin, Dongxu Liu, Fulan Wei

**Affiliations:** 1 Department of Orthodontics, Shandong Provincial Key Laboratory of Oral Tissue Regeneration, School of Stomatology, Shandong University, Wenhua Xi Road No. 44-1, Jinan, Shandong 250012 People’s Republic of China; 2 Shandong Provincial Key Laboratory of Oral Tissue Regeneration, School of Stomatology, Shandong University, Jinan, People’s Republic of China

**Keywords:** Long non-coding RNAs, Circular RNA, Competitive endogenous RNA, Periodontal ligament stem cells, Osteogenic differentiation, RNA sequencing

## Abstract

**Background:**

Researchers have been exploring the molecular mechanisms underlying the control of periodontal ligament stem cell (PDLSC) osteogenic differentiation. Recently, long noncoding RNAs (lncRNAs) and circular RNAs (circRNAs) were shown to function as competitive endogenous RNAs (ceRNAs) to regulate the effect of microRNAs (miRNAs) on their target genes during cell differentiation. However, comprehensive identification and integrated analysis of lncRNAs and circRNAs acting as ceRNAs during PDLSC osteogenic differentiation have not been performed.

**Results:**

PDLSCs were derived from healthy human periodontal ligament and cultured separately with osteogenic induction and normal media for 7 days. Cultured PDLSCs were positive for STRO-1 and CD146 and negative for CD31 and CD45. Osteo-induced PDLSCs showed increased ALP (alkaline phosphatase) activity and up-regulated expression levels of the osteogenesis-related markers ALP, Runt-related transcription factor 2 and osteocalcin. Then, a total of 960 lncRNAs and 1456 circRNAs were found to be differentially expressed by RNA sequencing. The expression profiles of eight lncRNAs and eight circRNAs were measured with quantitative real-time polymerase chain reaction and were shown to agree with the RNA-seq results. Furthermore, the potential functions of lncRNAs and circRNAs as ceRNAs were predicted based on miRanda and were investigated using Gene Ontology and Kyoto Encyclopedia of Genes and Genomes analysis. In total, 147 lncRNAs and 1382 circRNAs were predicted to combine with 148 common miRNAs and compete for miRNA binding sites with 744 messenger RNAs. These mRNAs were predicted to significantly participate in osteoblast differentiation, the MAPK pathway, the Wnt pathway and the signaling pathways regulating pluripotency of stem cells. Among them, lncRNAs coded as TCONS_00212979 and TCONS_00212984, as well as circRNA BANP and circRNA ITCH, might interact with miRNA34a and miRNA146a to regulate PDLSC osteogenic differentiation via the MAPK pathway.

**Conclusions:**

This study comprehensively identified lncRNAs/circRNAs and first integrated their potential ceRNA function during PDLSC osteogenic differentiation. These findings suggest that specific lncRNAs and circRNAs might function as ceRNAs to promote PDLSC osteogenic differentiation and periodontal regeneration.

**Electronic supplementary material:**

The online version of this article (10.1186/s12863-017-0569-4) contains supplementary material, which is available to authorized users.

## Background

Periodontitis is a slowly progressive disease characterized by the loss of periodontal tissue, which is the main cause of tooth loss [1]. With the development of stem-cell delivery therapeutics, periodontal ligament stem cells (PDLSCs) have been shown to produce typical periodontal ligament-like tissue to regenerate tissue damaged by periodontitis [2, 3]. Our previous studies also have shown the great potential of PDLSCs to regenerate periodontal tissue and form a bioengineered tooth root in miniature pigs [4–6]. The regeneration potency of PDLSCs contributes to their self-renewal and multi-differentiation capacity, especially osteogenic differentiation [7]. Exploring the molecular mechanisms of PDLSC osteogenic differentiation might provide new genetic strategies for periodontal regenerative medicine.

Recently, through microarray analysis, microRNAs (miRNAs) were identified and predicted to be involved in PDLSC osteogenic differentiation [8]. In addition, miRNA146a, miRNA17 and miRNA22 have been demonstrated to regulate PDLSC osteogenic differentiation by modulating the expression of target genes at the post-transcriptional level [9–11]. However, recent studies have revealed that a new player, competing endogenous RNA (ceRNA), is essential for the circuitry of miRNAs and target genes [12]. By competing for common miRNA response elements (MREs), ceRNAs can break the balance between miRNAs and target genes to regulate the physiological and pathophysiological process [13]. These ceRNAs include various types of RNAs, such as long non-coding RNAs (lncRNAs), circular RNAs (circRNAs), messenger RNAs (mRNAs) and pseudogenes.

LncRNA is a class of non-coding RNA (ncRNA) transcripts longer than 200 nucleotides [14]. Recent studies have reported that lncRNAs were involved in the osteogenic differentiation process [15]. For example, up-regulation of lncRNA HIF 1α-anti-sense 1 induced by TGFβ-mediated targeting of sirtuin 1 promotes the osteogenic differentiation of human bone marrow stromal cells [16]. Furthermore, lncRNA ANCR was proved to be critical in regulating the PDLSC osteogenic differentiation via the Wnt signaling pathway [17].

CircRNA is another new class of RNA composed of more than one exon with a covalently closed loop [18]. Compared to linear RNA, this circular structure is more stable and resistant to RNase R [19]. Emerging evidence has revealed that circRNAs participate in osteogenic differentiation [20, 21]. For instance, 154 differentially expressed circRNAs were found to associate with bone morphogenetic protein 2-induced osteogenic differentiation of MC3T3-E1 cells [22]. Moreover, circRNAs were predicted to have potential roles in osteogenesis of PDLSCs [23].

Although several lncRNAs, circRNAs and miRNAs are suggested to be involved in PDLSC osteogenic differentiation, there is not much data published on their potential networks and functions. To fully understand the impact of ceRNA crosstalk on PDLSC osteogenic differentiation, it will be crucial to integrate the lncRNA/circRNA-miRNA-mRNA competitive regulatory networks. In this study, we developed RNA sequencing (RNA-seq) with Illumina HiSeq2000 to comprehensively identify differentially expressed lncRNAs and circRNAs in normal and osteogenic inductive PDLSCs. Subsequently, the representative lncRNAs and circRNAs were further confirmed using quantitative real-time polymerase chain reaction (qRT-PCR). Finally, we predicted the ceRNA networks of lncRNAs, circRNAs, miRNAs and mRNAs based on miRanda and investigated their potential regulatory roles via gene ontology (GO) and Kyoto Encyclopedia of Genes and Genomes (KEGG) analysis. Our findings might provide new evidence for exploring the molecular mechanism of PDLSC osteogenic differentiation.

## Methods

All protocols for the handling of human tissues were approved by the Research Ethics Committee of Stomatology Hospital of Shandong University, China (G201401601). Informed consent was obtained from all donors.

### Cell culture and osteogenic induction

In this study, PDLSCs were derived from the middle third of the root surface of normal human impacted third molars, which were collected from 18- to 20-year-old patients at the Department of Oral Maxillofacial Surgery, Stomatology Hospital of Shandong University, using the explant culture method. Then, they were cultured with normal media, consisting of α-modification of Eagle’s media (HyClone, South Logan, UT, USA), 10% fetal calf serum (Gibco BRL, Grand Island, NY, USA), 100 U/ml penicillin and 100 μg/ml streptomycin (Invitrogen, Carlsbad, CA, USA) at 37 °C in 5% carbon dioxide. The stemness of PDLSCs was characterized by the scanning of cell surface markers (STRO-1, CD146, CD31, and CD45) through flow cytometric analysis (Becton, Dickinson and Company, Franklin Lakes, NJ, USA). For osteogenic differentiation, PDLSCs were cultured with osteogenic inductive media supplemented with 10 nM dexamethasone, 10 mM β-glycerophosphate and 50 μg/ml vitamin C (Sigma-Aldrich, St. Louis, MO, USA). Through separately culture with osteogenic induction and normal media for 7 days, PDLSCs were divided into two groups: induced and non-induced groups. All cells in this study were used at passage number 3.

### Alkaline Phosphatase (ALP) staining and ALP activity assay

ALP staining and activity assay were performed using an Alkaline Phosphatase Kit (Sigma-Aldrich) as described previously [24]. Briefly, PDLSCs were fixed with 70% ethanol for 30 min and stained with a solution of sodium nitrite, FRV alkaline and naphthol AS-BI alkaline for 15 min away from light. For the ALP activity assays, total protein was isolated from two groups of PDLSCs, incubated for 15 min with citrate buffer and phosphatase substrate (Sigma-Aldrich), and then quantified by spectrophotometric absorbance at 405 nm.

### Quantitative real-time PCR (qRT-PCR)

Total RNA was isolated from two groups of PDLSCs. For analysis of linear transcripts, 1 μg of RNA per sample was reverse transcribed into cDNA using a cDNA Reverse Transcription Kit (Takara, Tokyo, Japan). Convergent primers were designed to detect lncRNAs and mRNAs. Divergent primers were designed to detect the circular form of circRNAs. Relative transcript levels were measured with quantitative PCR using a Roche LightCycler®480 sequence detection system (Roche, Basel, Switzerland) following the manufacturer’s protocol. Each 20 μl reaction volume contained 10 μl SYBR® Premix Ex Taq™ (Takara), 0.4 μl 10 μM forward primer (0.4 μM final), 0.4 μl 10 μM reverse primer (0.4 μM final), 200 ng of template cDNA and DEPC-treated water. GAPDH was used as an internal control to quantify and normalize the results. The primer pairs are listed in Additional file 1. The specificity of the reaction was determined by detection of the Tms of the amplification products immediately after the last reaction cycle. The 2^-△△CT^ value was used for comparative quantitation. All qRT-PCRs were performed in triplicate.

### Construction of cDNA libraries and high-throughput sequencing

Total RNA was extracted from two groups using Trizol (Invitrogen) according to the manufacturer’s protocols. Strand-specific cDNA libraries were constructed following a previously described protocol [25] and were sequenced using an Illumina HiSeq2000 sequencer (LC Biotech, Hangzhou, China) by performing a paired-end run with a 100 bp read length. The raw reads were processed by removing the adaptor reads and low-quality tags. All subsequent analyses were performed using clean reads.

### Identification of differentially expressed lncRNAs, circRNAs and mRNAs

The clean reads from two cDNA libraries were mapped to the human genome sequence in GENCODE Release 19 using TopHat version 2.0.9 [26]. The transcripts were then assembled and annotated using Cufflinks [27]. According to the annotation of the human genome sequence, the known lncRNAs and mRNAs were identified. The coding potential of the remaining unknown transcripts was calculated with a coding potential calculator based on the quality, completeness, and sequence similarity of the open reading frame to the proteins in the protein databases [28]. The remaining unknown transcripts of more than 200 base pairs (bp) were considered novel lncRNAs with a coding potential score of less than −1, which suggested that a transcript had no protein-coding capacity. The expression levels of lncRNAs and mRNAs were quantified as FPKM (fragments per kilobase of exon per million fragments mapped) using the Cuffdiff program [27]. The differential expression of lncRNAs and mRNAs was determined using DESeq, with *P* < 0.05 and fold change (FC) ≥ 2 [29].

Compared with linear RNAs, circRNAs exhibit distinct patterns of alternative back-splicing and alternative splicing. An upgraded computational pipeline (CIRCexplorer2) was used to systematically identify and annotate circRNAs [30]. The expression levels of circRNAs were quantified as RPM (mapped backsplicing junction reads per million mapped reads) using the EBSeq package [31]. The differential expression of circRNAs was determined with *P* < 0.05, FC ≥ 2, and circRNA junction reads ≥5 [31].

### Functional analysis

The ceRNA networks among lncRNAs, circRNAs, miRNAs and mRNAs were predicted based on miRanda with a maximum binding free energy of −20 [32]. First, we predicted and selected miRNAs binding with both differentially expressed lncRNAs and circRNAs. Then, target mRNAs of these selected miRNAs were predicted and compared to the differentially expressed mRNAs that were identified in the RNA-seq results. Subsequently, we selected the intersecting elements of target mRNAs and differentially expressed mRNAs to analyze their potential functions through GO functional annotation and KEGG pathway analysis. GO terms were enriched using Blast2GO [33] by referring to GO databases. Meanwhile, KEGG pathway analysis was performed by referring to KEGG pathway databases. Cytoscape3.5.1 was used to display the lncRNA/circRNA-miRNA-mRNA networks.

### Statistical analysis

Quantitative qRT-PCR datasets are presented as the means ± standard deviation (SD) of at least three independent experiments. The statistical calculations were performed with SPSS statistics software version 17.0. Student’s t-test was performed for normally distributed data to determine statistical significance. A *P*-value less than 0.05 was considered statistically significant.

## Results

### Identification and Osteogenic differentiation of PDLSCs

**Fig. 1:**
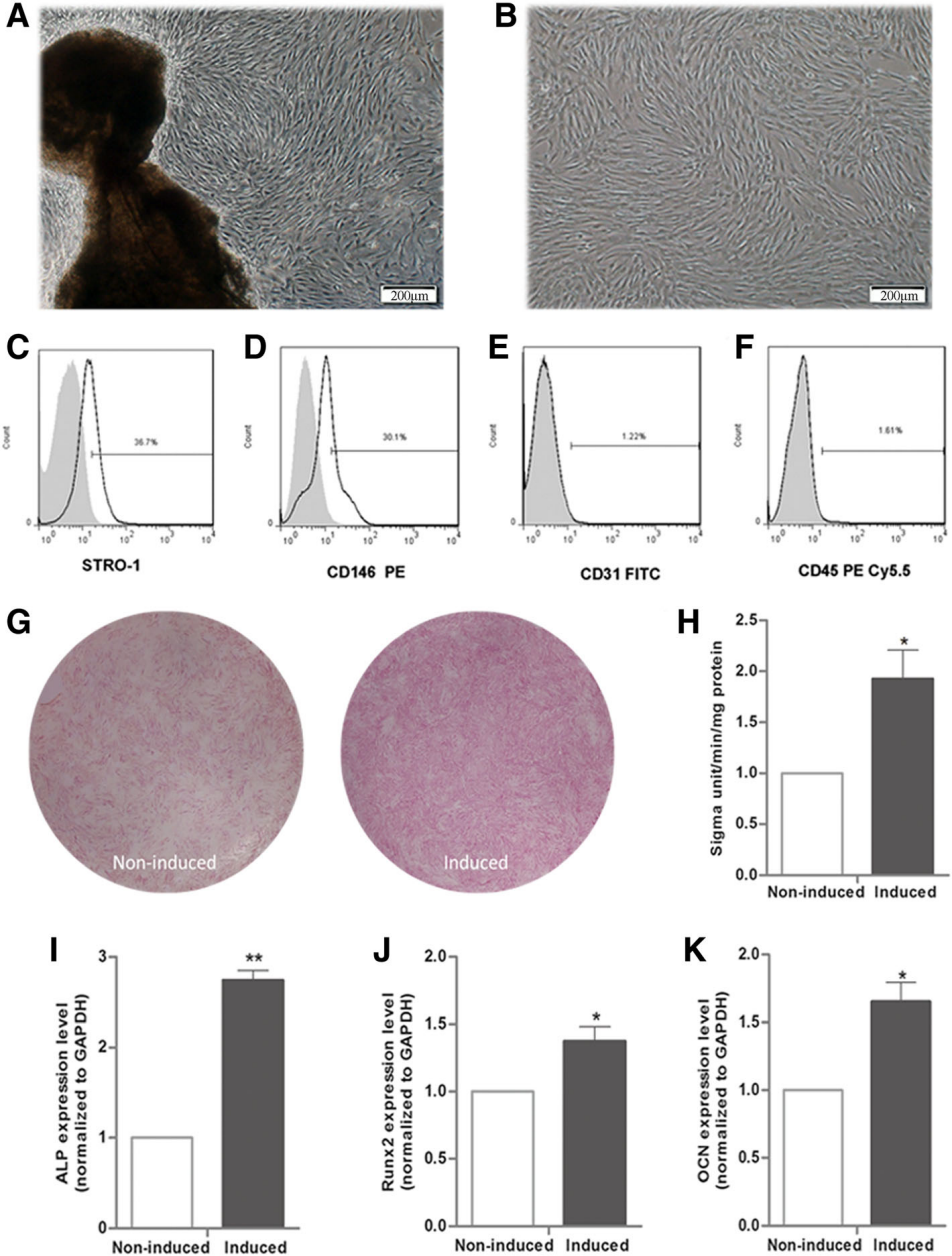
Identification and osteogenic differentiation of PDLSCs. **a** PDLSCs were derived from periodontal ligament explants. **b** PDLSCs were cultured with non-induced media at passage number 3. **c-f** PDLSCs were positive for STRO-1 and CD146 and negative for CD31 and CD45. **g, h** ALP activity was enhanced in osteo-induced PDLSCs (Induced), as evidenced by ALP staining and ALP activity assay. **i-k** Compared with the non-induced group, the induced group showed up-regulated expression of the osteogenic genes ALP, Runx2 and OCN by qRT-PCR. All PCRs were performed in triplicate. The data are represented as means ± SD. *, *P* < 0.05; **, *P* < 0.01

**Fig. 2:**
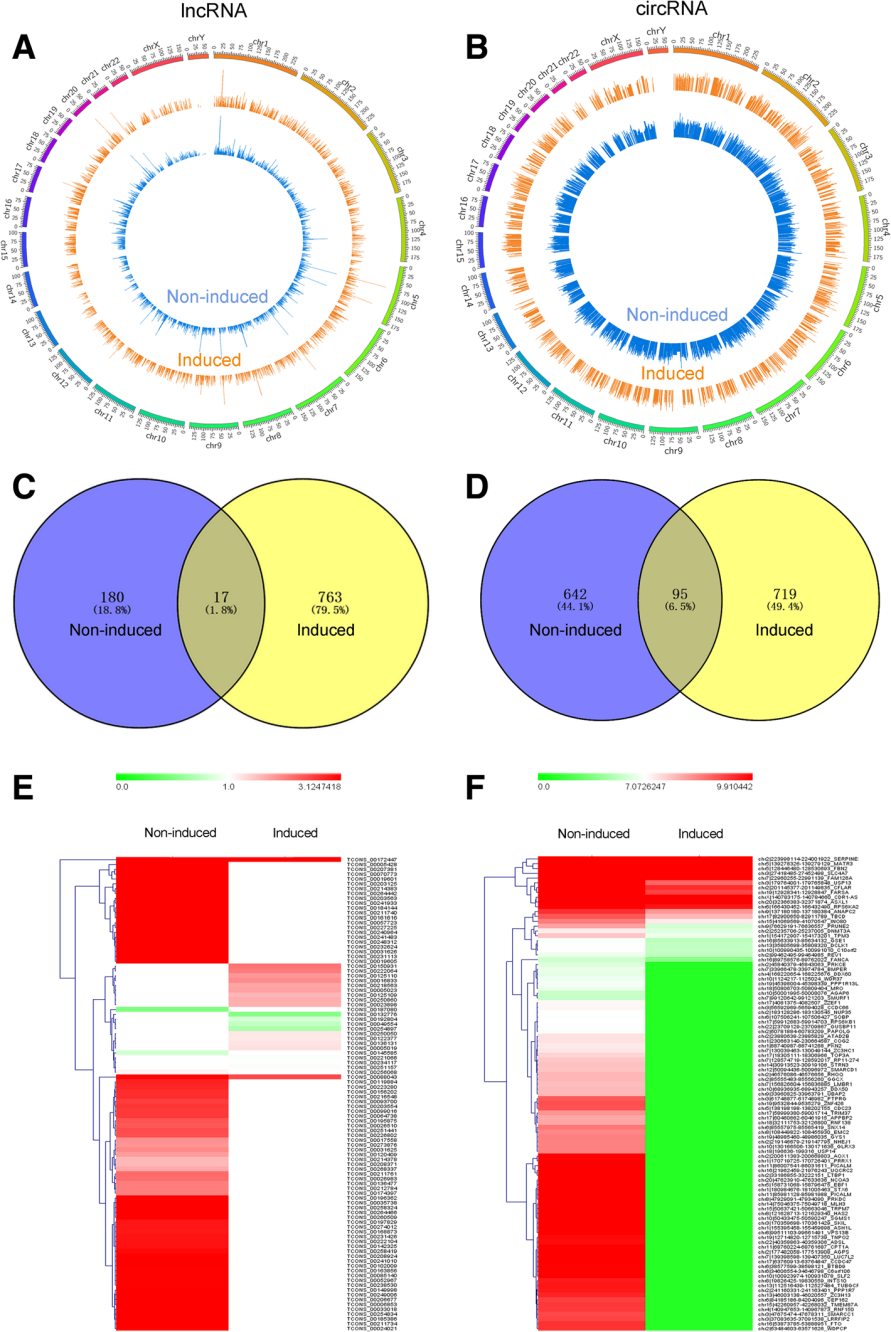
The apparent variations of differentially expressed lncRNAs and circRNAs**. (a, b)** LncRNAs and circRNAs were broadly distributed across the 24 pairs of human chromosomes according to their locations. The inner blue ring corresponds to the non-induced group; the outer yellow ring corresponds to the induced group. **c** Among differentially expressed lncRNAs, 17 common lncRNAs and 180 specific lncRNAs in the non-induced group and 763 specific lncRNAs in the induced group were identified. **d** Among differentially expressed circRNAs, 95 common circRNAs and 642 specific circRNAs in the non-induced group and 719 specific circRNAs in the induced group were identified. **e** Differentially expressed lncRNAs, consisting of 777 up-regulated lncRNAs and 183 down-regulated lncRNAs, are displayed in the heatmap. **f** Differentially expressed circRNAs, consisting of 766 up-regulated circRNAs and 690 down-regulated circRNAs, are displayed in the heatmap

PDLSCs derived from periodontal ligament explants were cultured with normal media to passage number 3 (Fig. 1a, b). Cultured PDLSCs were positive for STRO-1 and CD146 and negative for CD31 and CD45 (Fig. 1c-f). Increased ALP activity identified via ALP staining and ALP activity assay indicated osteogenic differentiation of osteo-induced PDLSCs (Fig. 1g, h). Subsequently, the up-regulated expression levels of the osteogenesis-related markers ALP, Runt-related transcription factor 2 (Runx2) and osteocalcin (OCN) provided further proof for the occurrence of PDLSC osteogenic differentiation (Fig. 2i-k). These findings agreed with previous reports on PDLSC differentiation into osteoblasts [7].

### Differential expression of lncRNAs, circRNAs and mRNAs upon osteogenic differentiation of PDLSCs

Ribosome-depleted RNA-seq generated 91.4 million raw reads of the non-induced group and 113.8 million raw reads of the induced group (Table 1). After filtering the adaptor reads and low-quality tags, we separately obtained 90.9 million and 112.4 million clean reads. More than 98% of the raw reads per sample were clean reads. According to the annotation of the human genome sequence GENCODE Release 19, 11,529 lncRNAs (non-induced group) and 9511 lncRNAs (induced group) were identified from the two cDNA libraries (Table 2) (Additional file 2). We also identified 5913 circRNAs in the non-induced group and 3162 circRNAs in the induced group (Table 2) (Additional file 3). In addition, 66,134 mRNAs (non-induced group) and 52,227 mRNAs (induced group) were annotated (Table 2) (Additional file 4). Both lncRNAs and circRNAs were broadly distributed across the 24 pairs of human chromosomes (Fig. 2a, b). A total of 960 lncRNAs, 1161 circRNAs and 1887 mRNAs were found to be differentially expressed, with *P*-value <0.05 and FC ≥ 2 (Additional file 5, Additional file 6 and Additional file 7). Among the differentially expressed lncRNAs, 17 common lncRNAs and 180 specific lncRNAs in the non-induced group and 763 specific lncRNAs in the induced group were detected, with 777 up-regulated lncRNAs and 183 down-regulated lncRNAs (Fig. 2c). Meanwhile, we also identified 95 common circRNAs and 642 specific circRNAs in the non-induced group and 719 specific circRNAs in the induced group, with 766 up-regulated circRNAs and 690 down-regulated circRNAs (Fig. 2d). The apparent variations in transcripts between the two groups are visually displayed with heatmaps (Fig. 2e, f).

**Table 1:** Statistical data of RNA-Seq reads for two samples

Sample	Raw reads	Clean reads	Unique lncRNAs	Unique circRNAs	Unique mRNAs
Non-induced	91.4 million	90.9 million	11,529	5913	66,134
Induced	113.8 million	112.4 million	9511	3162	52,227

**Table 2:** Expression profiles of lncRNAs validated by RNA-seq

Test_id.	Induced (FPKM)	Non-induced (FPKM)	Regulation
TCONS_00019601	93.6452	0	up
TCONS_00227764	830.447	47.6825	up
TCONS_00085268	0	26.6246	down
TCONS_00254538	18,683.1	3962.3	up
TCONS_00198784	9.25995	0.0230801	up
TCONS_00136898	8.67356	0.0105046	up
TCONS_00125934	0	4.65289	down
TCONS_00115113	0	4.05314	down

### Differentially expressed lncRNAs/circRNAs validated by qRT-PCR

**Fig. 3:**
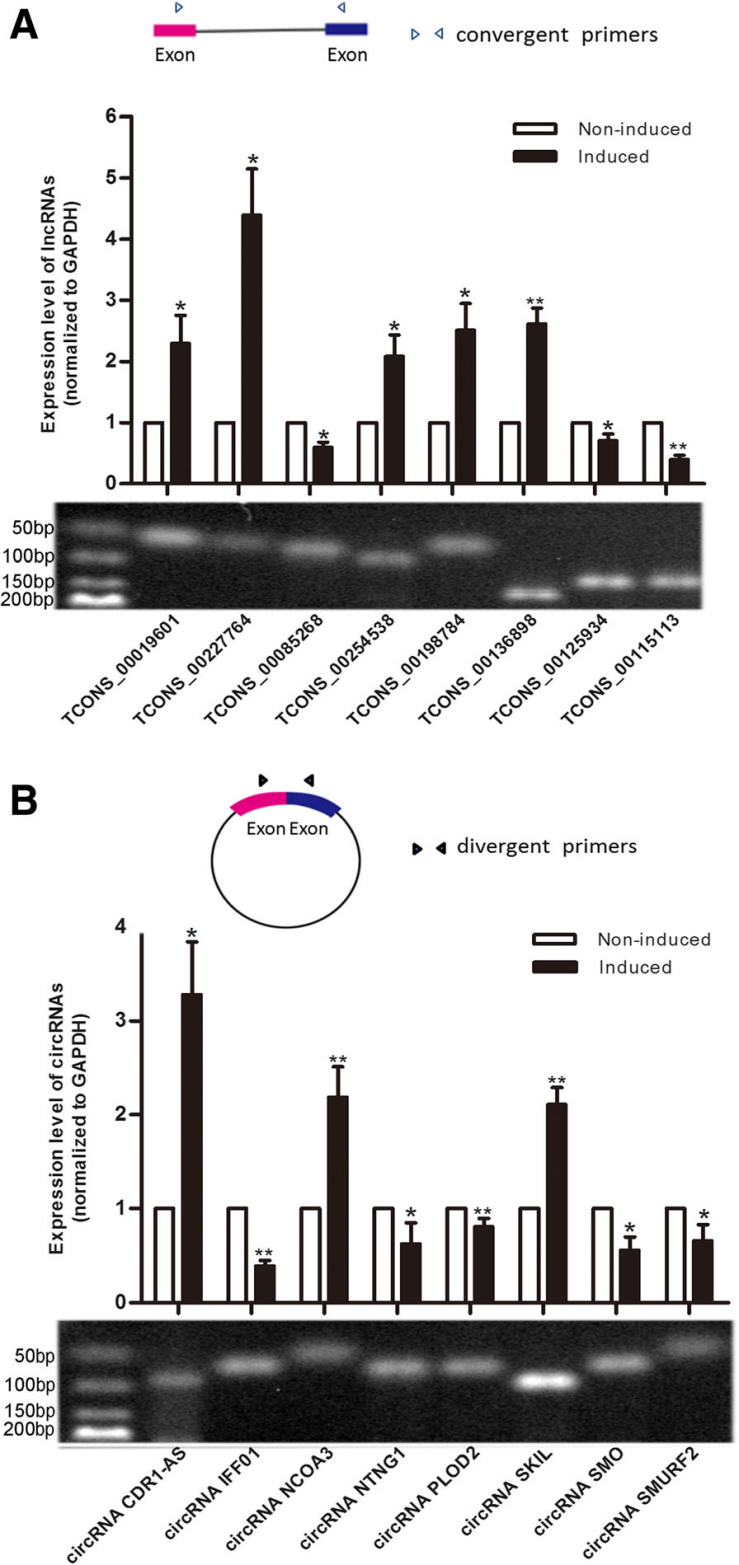
Differentially expressed lncRNAs/circRNAs validated by qRT-PCR**. a** Convergent primers were designed to detect eight lncRNAs with *P* < 0.05, FC ≥ 2 and FPKM in at least one of the samples ≥4. LncRNAs coded as TCONS_00019601, TCONS_00227764, TCONS_00254538, TCONS_00198784 and TCONS_00136898 were up-regulated in the induced group compared with the non-induced group, and TCONS_00085268, TCONS_00125934, and TCONS_00115113 were down-regulated. **b** Divergent primers were designed to detect the circular form of circRNAs with *P* < 0.05, FC ≥ 2 and RPM in at least one of the samples ≥4. CircRNAs named CDR1-AS, NCOA3, and SKIL were up-regulated in the induced group compared to the non-induced group, and the circRNAs IFF01, NTNG1, PLOD2, SMO, and SMURF2 were down-regulated. The results agreed with the normalized expression of validated lncRNAs and circRNAs shown in Tables 2 and 3. All PCRs were performed in triplicate. The data are represented as means ± SD. *, *P* < 0.05; **, *P* < 0.01

To verify the results of the RNA-seq experiments, eight lncRNAs and eight circRNAs with *P* < 0.05, FC ≥ 2 and FPKM/RPM in at least one of the samples ≥4 were selected for qRT-PCR validation. The lncRNAs were amplified with convergent primers and the circRNAs were amplified with divergent primers (Fig. 3a, b). Compared to the non-induced group, the induced group showed increased expression of the lncRNAs coded as TCONS_00019601, TCONS_00227764, TCONS_00254538, TCONS_00198784 and TCONS_00136898 and decreased expression of TCONS_00085268, TCONS_00125934, and TCONS_00115113 (Fig. 3a). The circRNAs named CDR1-AS, NCOA3, and SKIL were up-regulated in the induced group compared to the non-induced group, and the circRNAs IFF01, NTNG1, PLOD2, SMO, and SMURF2 were down-regulated (Fig. 3b). All the results were consistent with the normalized expression of RNA-seq shown in Tables 2 and 3.

**Table 3:** Expression profiles of circRNAs validated by RNA-seq

Name	Induced (RPM)	Non-induced (RPM)	Regulation
circRNA CDR1-AS	3038.695	1102.345	up
circRNA IFF01	0	337.2772	down
circRNA NCOA3	1421.615	0	up
circRNA NTNG1	0	1286.478	down
circRNA PLOD2	0	3742.131	down
circRNA SKIL	1956.098	0	up
circRNA SMO	0	783.6154	down
circRNA SMURF2	0	282.7267	down

### Function of lncRNAs and circRNAs as ceRNAs via lncRNA/circRNA-miRNA-mRNA networks

**Fig. 4:**
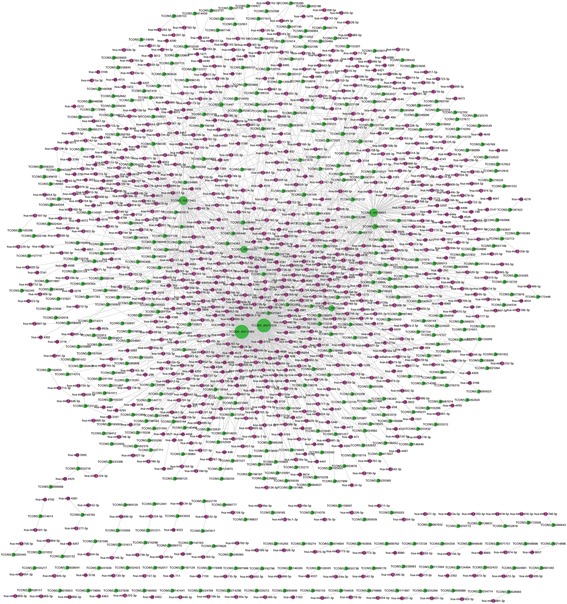
CeRNA networks of 430 lncRNAs and 779 miRNAs based on miRanda

**Fig. 5:**
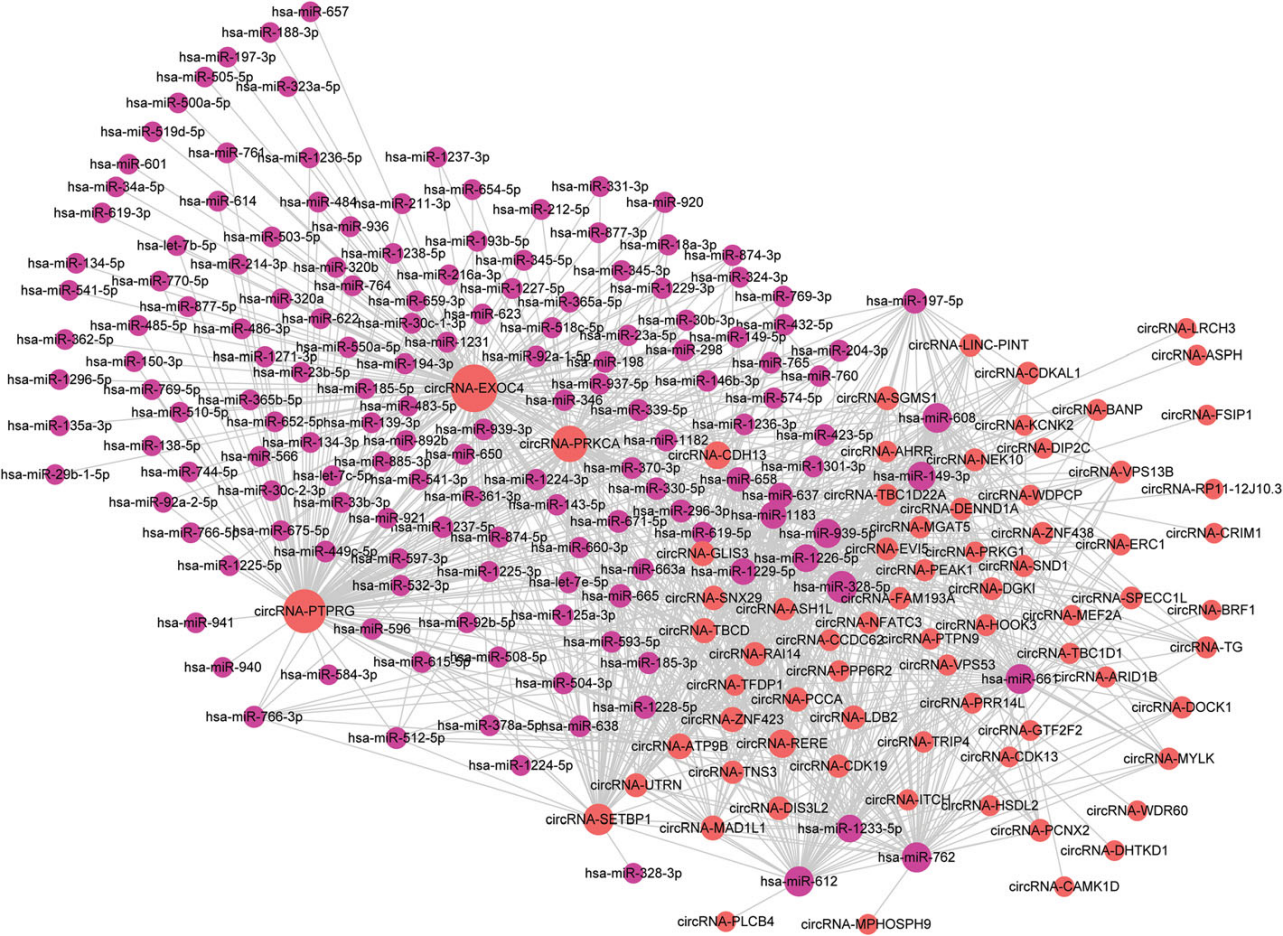
CeRNA networks of selected circRNAs and miRNAs based on miRanda. Considering that graphics cannot display the enormous amount of network information between 1401 circRNAs and 855 miRNAs, we selected circRNAs with more miRNA binding sites and less binding free energy to make the network diagram using Cytoscape3.5.1

Based on miRanda with the maximum binding free energy of −20, 430 lncRNAs were predicted to share at least two binding sites with 779 miRNAs (Fig. 4) (Additional file 8). We also predicted that 1401 circRNAs bind 855 miRNAs with at least two binding sites (Additional file 9). Considering that graphics cannot display the enormous amount of network information between 1401 circRNAs and 855 miRNAs, we selected circRNAs with more miRNA binding sites and less binding free energy to make the network diagram (Fig. 5). Through analysis of the common binding MREs of lncRNAs and circRNAs, 165 miRNAs were predicted to combine with both 158 lncRNAs and 1385 circRNAs (data not shown).

**Fig. 6:**
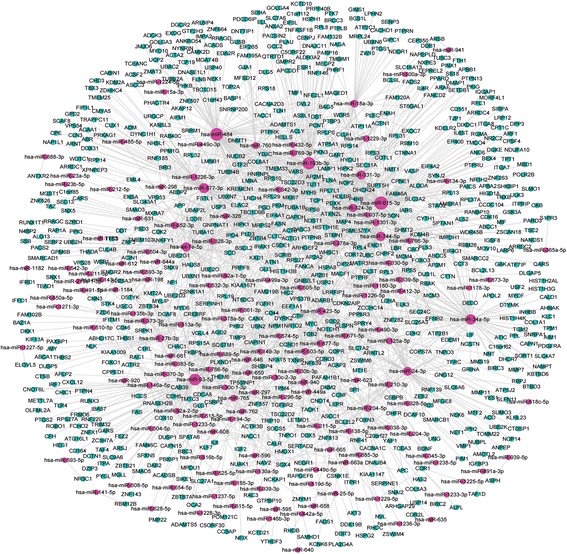
CeRNA networks of 744 mRNAs and 148 common miRNAs based on miRanda

To reveal their potential function, we predicted target mRNAs of these miRNAs based on miRanda and compared these target mRNAs to the differentially expressed mRNAs that were identified in the RNA-seq results (Additional file 7). There were 744 differentially expressed mRNAs that were found to combine with 148 common miRNAs along with 147 lncRNAs and 1382 circRNAs (Additional file 10). The networks of 744 mRNAs and 148 common miRNAs are shown in Fig. 6.

**Fig. 7:**
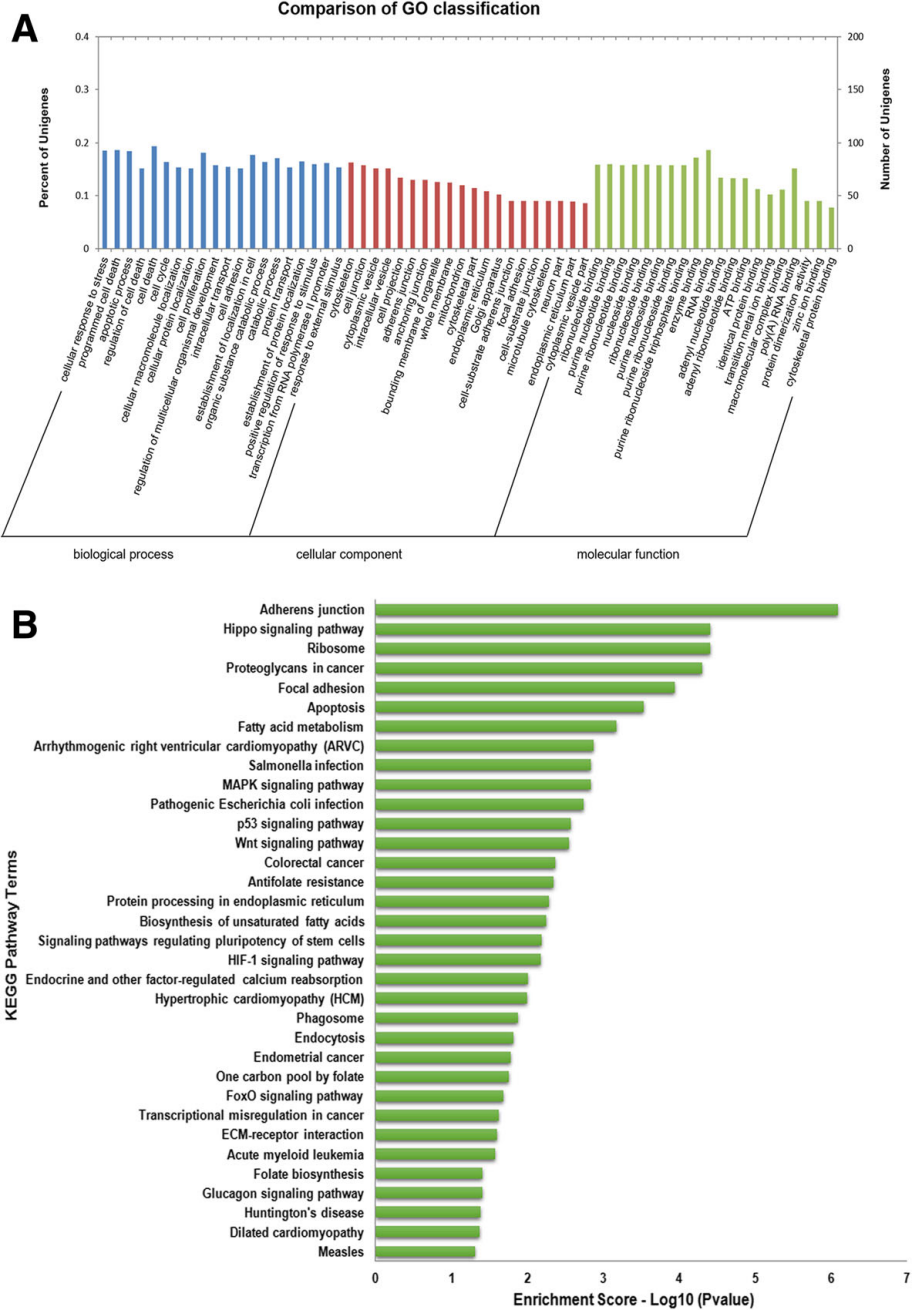
GO annotations and KEGG pathway analysis of 744 differentially expressed mRNAs**. a** GO annotations (P < 0.05) involving the top sixty numbers of mRNAs included multiple biological processes, cellular components and molecular functions. **b** These mRNAs were significantly enriched in 34 KEGG pathways, including the the MAPK pathway, the Wnt pathway and the signaling pathways regulating pluripotency of stem cells

**Fig. 8:**
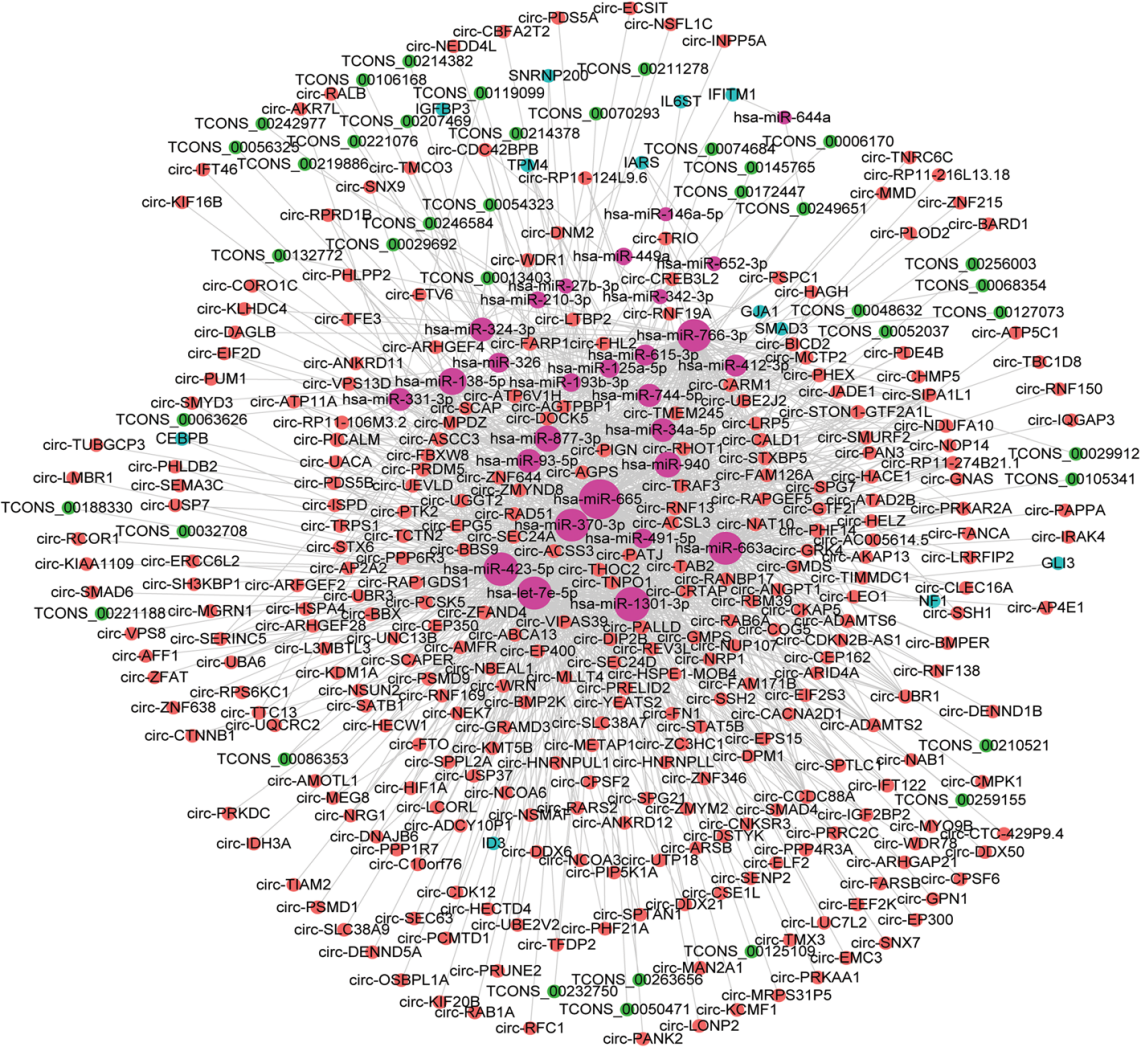
CeRNA networks of lncRNAs/circRNAs-miRNAs-mRNAs significantly participated in GO: 0001649 (osteoblast differentiation)

The potential regulatory roles of the ceRNA networks were predicted by analyzing the functions of 744 mRNAs through GO and KEGG pathway analysis (Additional file 11 and Additional file 12). GO annotations (*P* < 0.05) involving the top 60 mRNAs are displayed in Fig. 7a and include multiple biological processes, cellular components and molecular functions. Among these GO terms, we obtained GO: 0001649 (osteoblast differentiation), which was significantly enriched by 21 mRNAs (Additional file 11). The complex mRNA networks involved in GO: 0001649 (osteoblast differentiation) and related miRNAs, lncRNAs, and circRNAs are displayed in Fig. 8. Through KEGG analysis, mRNAs were predicted to participte in 34 pathways (Fig. 7b). Among these KEGG pathways, the MAPK pathway, the Wnt pathway and the signaling pathways regulating pluripotency of stem cells were closely related to osteogenic differentiation.

**Fig. 9:**
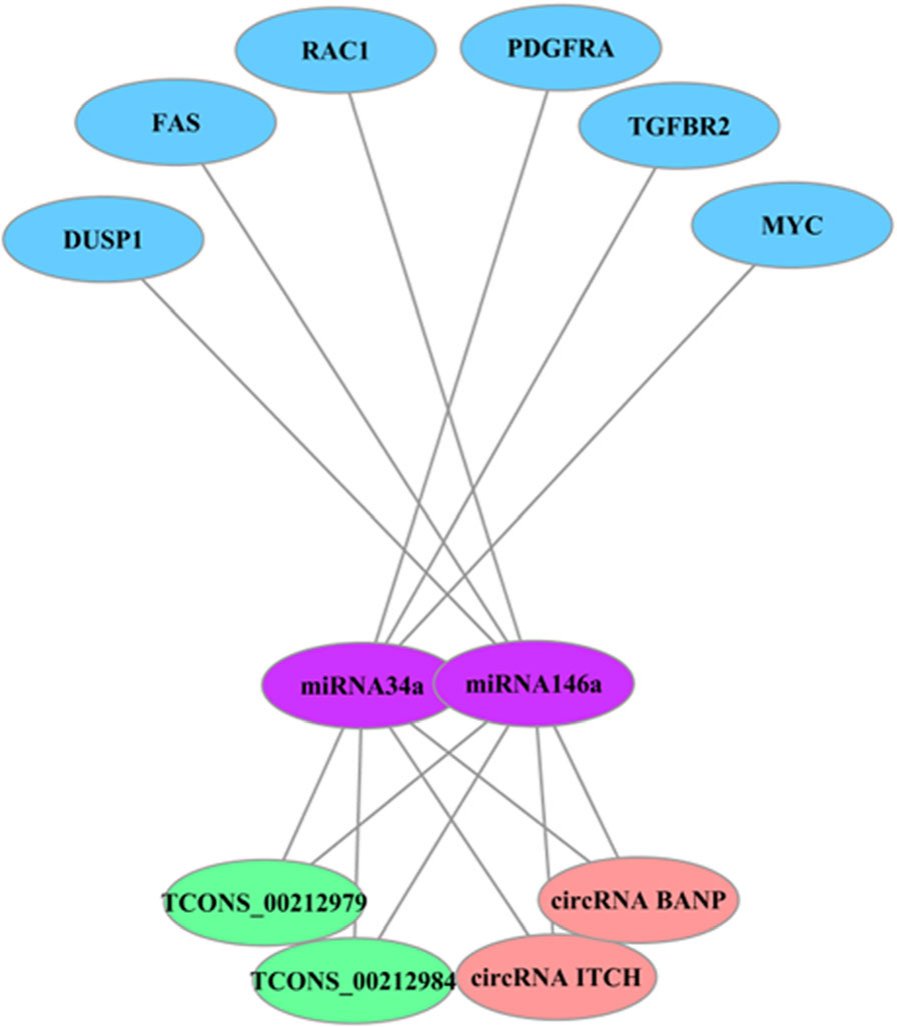
CeRNA networks of lncRNAs/circRNAs-miRNAs-mRNAs significantly participated in the MAPK pathway**.** The lncRNAs coded as TCONS_00212979 and TCONS_00212984, as well as circRNA BANP and circRNA ITCH, were predicted to interact with miRNA34a and miRNA146a. DUSP1, FAS and RAC1 were predicted to be target genes of miRNA34a. PDGFRA, TGFBR2 and MYC were predicted to be targeted by miRNA146a. These six mRNAs are pivotal genes in the MAPK pathway according to KEGG analysis

Based on the above results, we selected several lncRNAs, circRNAs, miRNAs and mRNAs associated with the MAPK pathway to further display the ceRNA networks (Fig. 9). The lncRNAs coded as TCONS_00212979 and TCONS_00212984, circRNA BANP and circRNA ITCH were predicted to combine with miRNA34a and miRNA146a. DUSP1, FAS and RAC1 were predicted to be target genes of miRNA34a. In addition, PDGFRA, TGFBR2 and MYC were predicted to be targeted by miRNA146a. These six mRNAs were the pivotal genes of the MAPK pathway according to the KEGG analysis. This complicated ceRNA network suggested that TCONS_00212979, TCONS_00212984, circRNA BANP and circRNA ITCH might play regulatory roles in the MAPK pathway through miRNA34a, miRNA146a and their targets during PDLSC osteogenic differentiation.

## Discussion

LncRNAs, circRNAs, miRNAs and mRNAs form large-scale ceRNA cross-talk networks through MREs, which has exciting implications for gene regulation at the post-transcriptional level during multiple physiological and pathophysiological processes [12, 34]. In recent years, studies have documented the functions and clinical implications of ceRNAs in cancer, systemic lupus erythematosus and other diseases, which may present opportunities for new therapeutic approaches for diseases [35, 36].

Recently, researchers have systematically constructed ceRNA networks through RNA-seq and bioinformatics in mouse germline stem cells to reveal functions and mechanisms of lncRNAs and circRNAs in mouse germline stem cell self-renewal and differentiation [37]. Moreover, lncRNA POIR was demonstrated to form a regulatory network with miRNA182 and FoxO1 to up-regulate PDLSC osteogenic differentiation in periodontitis patients [38]. However, the ceRNA networks were revealed to be intertwined [39]. To fully understand the impact of ceRNA crosstalk on PDLSC osteogenic differentiation, it will be crucial to integrate the competitive lncRNA/circRNA-miRNA–mRNA regulatory networks. In our study, 744 mRNAs were predicted to combine with 148 common miRNAs, along with 147 lncRNAs and 1382 circRNAs.

Through GO analysis, 21 mRNAs were predicted to significantly participate in osteoblast differentiation (GO: 0001649) (Fig. 8). Among them, ALPL, also called ALP, was reported to be an osteogenesis-related marker and was up-regulated during PDLSC osteogenic differentiation [7]. The up-regulated expression level of ALP was also detected by qRT-PCR in our study (Fig. 1i). SMAD3 and SMAD5, members of the SMAD family, were also predicted to form ceRNA networks and participate in osteoblast differentiation. Both SMAD3 and SMAD5 were demonstrated to regulate PDLSC osteogenic differentiation by modulating TGF-β signals [40, 41]. Additionally, Notch1, another participant in osteoblast differentiation (GO: 0001649), is part of the Notch signaling pathway, which is important for maintaining osteogenic differentiation of PDLSCs [42, 43]. These crucial osteogenic genes formed ceRNA networks with lncRNAs and circRNAs by targeting common miRNAs, and these networks might provide evidence of new regulatory mechanisms in PDLSC osteogenic differentiation.

Through KEGG pathway analysis, mRNAs of ceRNA networks were predicted to be involved in the Wnt pathway, MAPK pathway and signaling pathways regulating the pluripotency of stem cells. Previous studies have demonstrated that Wnt signaling contributes to the differentiation of periodontal ligament fibroblasts into osteoblasts [44]. In addition, the MAPK pathway was found to be critical for skeleton development and bone homeostasis [45]. Moreover, it plays significant roles in osteogenic differentiation of PDLSCs [46, 47]. Furthermore, we constructed a ceRNA network of TCONS_00212979, TCONS_00212984, circRNA BANP, circRNA ITCH, miRNA34a, miRNA146a, DUSP1, FAS, RAC1, PDGFRA, TGFBR2 and MYC. These mRNAs are important elements of the MAPK pathway based on KEGG analysis. Among them, DUSP1, FAS and RAC1 are targeted by miRNA34a, while PDGFRA, TGFBR2 and MYC are targeted by miRNA146a. Studies have illustrated that both miRNA34a and miRNA146a are closely related to osteogenic differentiation of human mesenchymal stem cells [48, 49]. In addition, miRNA146a was revealed to promote differentiation of periodontal ligament cells [9]. TCONS_00212979, TCONS_00212984, circRNA BANP and circRNA ITCH were predicted to bind miRNA34a and miRNA146a. TCONS_00212979, known as CARMEN, has been reported to be a cardiac mesoderm enhancer-associated lncRNA that modulates cardiac differentiation through miRNA143 and miRNA145 [50]. TCONS_00212984 is a novel lncRNA with a genomic origin similar to that of TCONS_00212979 according to RNA-seq. circRNA BANP and circRNA ITCH have both been reported to contribute to carcinogenesis and might serve as cancer biomarkers [51, 52]. However, the regulatory roles of these two lncRNAs and circRNAs in osteogenic differentiation remain unclear. In summary, the ceRNA network suggested that TCONS_00212979, TCONS_00212984, circRNA BANP and circRNA ITCH might interact with miRNA34a and miRNA146a to regulate PDLSC osteogenic differentiation via the MAPK pathway. However, their regulatory mechanisms need to be further investigated. Our future study plan will be to validate their differential expression profiles, verify their ceRNA networks and specify their effects on PDLSC osteogenic differentiation using knockdown and overexpression experiments.

## Conclusion

This study identified differentially expressed lncRNAs, circRNAs and mRNAs during osteogenic differentiation of PDLSCs. Competitive lncRNA/circRNA-miRNA–mRNA regulatory networks were comprehensively integrated and predicted to be involved in osteoblast differentiation by GO and KEGG pathway analysis. Moreover, the lncRNAs coded as TCONS_00212979 and TCONS_00212984, circRNA BANP and circRNA ITCH were predicted to interact with miRNA34a and miRNA146a to regulate PDLSC osteogenic differentiation via the MAPK pathway. Our study suggested that specific lncRNAs and circRNAs might function as ceRNAs to promote PDLSC osteogenic differentiation and periodontal regeneration.

## Additional files

Additional file 1: Gene Primers.doc Gene primers used in qRT-PCR (DOC 38 kb)

Additional file 2: lncRNA_all expression_.xlsx The expression profiles of all lncRNAs identified by RNA-seq (XLSX 1653 kb)

Additional file 3: circRNA_all expression_.xlsx The expression profiles of all circRNAs identified by RNA-seq (XLSX 1577 kb)

Additional file 4: mRNA_all expression_.xlsx The expression profiles of all mRNAs identified by RNA-seq (XLSX 8078 kb)

Additional file 5: lncRNA_differential expression.xlsx Differentially expressed lncRNAs identified by RNA-seq (XLSX 87 kb)

Additional file 6: circRNA_differential expression.xlsx Differentially expressed circRNAs identified by RNA-seq (XLSX 312 kb)

Additional file 7: mRNA_differential expression.xlsx Differentially expressed mRNAs identified by RNA-seq (XLSX 240 kb)

Additional file 8: lncRNA_miRNA_miRanda_2BindingSites.xlsx LncRNAs predicted to share at least two miRNA binding sites (XLSX 115 kb)

Additional file 9: circRNA_miRNA_miRanda_2BindingSites.xlsx CircRNAs predicted to share at least two miRNA binding sites (XLSX 23183 kb)

Additional file 10: lncRNA-circRNA-miRNA-mRNA.xlsx LncRNAs and circRNAs predicted to share miRNA binding sites in common with target mRNAs (XLSX 8696 kb)

Additional file 11: target mRNAs_GO enrichment.xlsx GO items enriched by target mRNAs through GO analysis (XLSX 95 kb)

Additional file 12: target mRNAs_KEGG pathway enrichment.xlsx Pathways enriched by target mRNAs through KEGG analysis (XLSX 20 kb)

## Abbreviations

ALP: Alkaline phosphatase
bp: Base pairs
ceRNAs: Competitive endogenous RNAs
circRNAs: Circular RNAs
FC: Fold change
FPKM: Fragments per kilobase of exon per million fragments mapped
GO: Gene Ontology
KEGG: Kyoto Encyclopedia of Genes and Genomes
lncRNAs: Long non-coding RNAs
miRNAs: MicroRNAs
MREs: miRNA response elements
mRNAs: Messenger RNAs
ncRNA: Non-coding RNA
OCN: Osteocalcin
PDLSC: Periodontal ligament stem cell
qRT-PCR: Quantitative real-time polymerase chain reaction
RNA-seq: RNA sequencing
RPM: Mapped back-splice junction reads per million mapped reads
Runx2: Runt-related transcription factor 2
SD: Standard deviation
