# Supplementary material for: Identification and integrated analysis of differentially expressed lncRNAs and circRNAs reveal the potential ceRNA networks during PDLSC osteogenic differentiation
Source: BMC Genet. 2017 Dec 2;18:100. doi: 10.1186/s12863-017-0569-4 (PMC5712120; doi:10.1186/s12863-017-0569-4)
Supplement: Supplementary file 1 — Gene Primers.doc Gene primers used in qRT-PCR (DOC 38 kb) [file 12863_2017_569_MOESM1_ESM.doc]

Gene Primers

| test_id or gene name | Forward primer(5'-3') | Reverse primer（5'-3') |
| --- | --- | --- |
| GAPDH | TCATGGGTGTGAACCATGAGAA | GGCATGGACTGTGGTCATGAG |
| ALP | CCACGTCTTCACATTTGGTG | AGACTGCGCCTGGTAGTTGT |
| Runx2 | CGAATGGCAGCACGCTATTAA | GTCGCCAAACAGATTCATCCA |
| OCN | TAGTGAAGAGACCCAGGCGCT | ATAGGCCTCCTGAAAGCCGA |
| TCONS_00019601 | ACGTCGAGGGTATTGTGTCC | CAAGCCGCAAGACTAGGACG |
| TCONS_00227764 | CATTTGCGTTGCGTTCCGAT | CTGCCAAAGAAGTTGGATACAC |
| TCONS_00085268 | TATTGTGTCCCCTCTGGACG | AGACTAGGACGACAGAAGCG |
| TCONS_00254538 | CTCTCTTAAGGTAGCCAAATGCC | GGGACAGTGGGAATCTCGTT |
| TCONS_00198784 | GTAACAATTTTGCGCTTTGCTTCC | GGGTTGGGTTCCTGTCCCG |
| TCONS_00136898 | CCCCTGGAAAGAGCACACTG | TTCCGGGTTTGCTGTTTTGTC |
| TCONS_00125934 | CTGCCTACAAGCCTCACAGTCC | GGAATTGTCCCAACAGGGATGG |
| TCONS_00115113 | CGTGAAGCAAGTCCCACTGA | GACGGGGTTTTCCTTGTCCT |
| circRNA CDR1-AS | TGACATTCAGGTCTTCCAGTGT | TTGACACAGGTGCCATCGGA |
| circRNA IFF01 | GCGCTCCCGCTTCCG | CAGCTGCAGAGGCCCAG |
| circRNA NCOA3 | GCAGTCATGGTCCCAGAAACG | CCCGTCTCCGTTTTTCACCAC |
| circRNA NTNG1 | AGACATAAAGGTGCGAGGAAGG | TTGCACATGTAGGGATTGCC |
| circRNA PLOD2 | ATCACTTTCTTTTGTTGCTACAGTT | GGCAATTAATGGAAATGGACCC |
| circRNA SKIL | AGGAGAAGTTTAGCATGAGAAGTG | AATTGCCCAGTTATCTTCAAGGATT |
| circRNA SMO | ATCGTGGGAGGCTACTTCCT | ACTGGCCTGAACTGTTGAACT |
| circRNA SMURF2 | TTGCACAGAGTAAGGTCATAATGC | TGATCCAAAGTGGAATCAGCA |
